# Pancreatic Alpha-Cell Dysfunction Contributes to the Disruption of Glucose Homeostasis and Compensatory Insulin Hypersecretion in Glucocorticoid-Treated Rats

**DOI:** 10.1371/journal.pone.0093531

**Published:** 2014-04-04

**Authors:** Alex Rafacho, Luiz M. Gonçalves-Neto, Junia C. Santos-Silva, Paloma Alonso-Magdalena, Beatriz Merino, Sebastião R. Taboga, Everardo M. Carneiro, Antonio C. Boschero, Angel Nadal, Ivan Quesada

**Affiliations:** 1 Department of Physiological Sciences, Center of Biological Sciences, Federal University of Santa Catarina (UFSC), Florianópolis, Brazil; 2 Department of Structural and Functional Biology, Institute of Biology, and Obesity and Comorbidities Research Center (OCRC), State University of Campinas (UNICAMP), Campinas, Brazil; 3 Institute of Bioengineering and the Biomedical Research Center in Diabetes and Associated Metabolic Disorders (CIBERDEM), Miguel Hernández University, Elche, Spain; 4 Department of Biology, Institute of Biosciences, Humanities and Exact Sciences, São Paulo State University (UNESP), São José do Rio Preto, Brazil; University of Santiago de Compostela School of Medicine - CIMUS, Spain

## Abstract

Glucocorticoid (GC)-based therapies can cause insulin resistance (IR), glucose intolerance, hyperglycemia and, occasionally, overt diabetes. Understanding the mechanisms behind these metabolic disorders could improve the management of glucose homeostasis in patients undergoing GC treatment. For this purpose, adult rats were treated with a daily injection of dexamethasone (1 mg/kg *b.w.*, *i.p.*) (DEX) or saline as a control for 5 consecutive days. The DEX rats developed IR, augmented glycemia, hyperinsulinemia and hyperglucagonemia. Treatment of the DEX rats with a glucagon receptor antagonist normalized their blood glucose level. The characteristic inhibitory effect of glucose on glucagon secretion was impaired in the islets of the DEX rats, while no direct effects were found on α-cells in islets that were incubated with DEX *in vitro*. A higher proportion of docked secretory granules was found in the DEX α-cells as well as a trend towards increased α-cell mass. Additionally, insulin secretion in the presence of glucagon was augmented in the islets of the DEX rats, which was most likely due to their higher glucagon receptor content. We also found that the enzyme 11βHSD-1, which participates in GC metabolism, contributed to the insulin hypersecretion in the DEX rats under basal glucose conditions. Altogether, we showed that GC treatment induces hyperglucagonemia, which contributes to an imbalance in glucose homeostasis and compensatory β-cell hypersecretion. This hyperglucagonemia may result from altered α-cell function and, likely, α-cell mass. Additionally, blockage of the glucagon receptor seems to be effective in preventing the elevation in blood glucose levels induced by GC administration.

## Introduction

Endogenous glucocorticoids (GCs), such as cortisol in humans and corticosterone in rodents, play a key role in several physiological functions like the regulation of glucose homeostasis and nutrient metabolism [1]. Exogenous synthetic compounds with GC activity, *e.g.*, prednisolone and dexamethasone, are broadly used for clinical purposes due to their anti-inflammatory, anti-allergic and immunosuppressive effects [2]. GC therapy provides beneficial effects for patients subjected to allotransplantation and for patients with rheumatoid diseases, bowel diseases or asthma, among other pathologies [2], [3]. However, when administered in excess or for long periods, GCs can cause several adverse effects [2], [3]. Development of glucose intolerance, insulin resistance (IR), hyperglycemia and dyslipidemia, especially among individuals who are more susceptible to these disorders, are among the diabetogenic effects of GC therapy [4]–[8].

Most of the adverse effects of GCs on metabolism are reversible upon the discontinuation of GC treatment [7], [9]. In addition, intermittent prescriptions and/or low doses of GCs may attenuate their diabetogenic effects [10]. However, patients receiving GCs are commonly subjected to prolonged therapy that may culminate in irreversible metabolic disorders, such as hyperglycemia or the onset of type 2 diabetes mellitus (T2DM) [1], [3]. Thus, glucose homeostasis should be controlled in patients undergoing chronic GC treatment or excessive acute exposure to steroid hormones.

A better understanding of the mechanisms involved in the steroid-induced impairment of glucose homeostasis is necessary to generate strategies to diminish the side effects of GC therapy. In this regard, GC-induced IR has been found to enhance β-cell function and mass, inducing hyperinsulinemia as a compensatory response to the increased insulin needs imposed by IR [6], [11]–[13]. When this compensation is not sufficient, hyperglycemia may arise [9], [12]. Impaired pancreatic α-cell function has emerged as an important factor in the etiology of the pathologies associated with the deregulation of glucose homeostasis, such as obesity [14], [15], T2DM [16], [17], and hypercortisolism [14]. In fact, a few animal [18] and human [14], [19] studies have reported that GC treatment may induce altered glucagon levels. However, the mechanism by which these changes occur has not been fully elucidated. Yet, given that glucagon modulates insulin release [20], [21], the potential contribution of altered glucagon levels to the β-cell hypersecretion and hyperinsulinemia of GC-treated subjects remains to be investigated.

To elucidate the involvement of glucagon in the disruption of glucose homeostasis and the insulin hypersecretion caused by GC treatment, we treated rats with dexamethasone for five consecutive days. These rats developed IR, hyperinsulinemia and mild hyperglycemia. We demonstrate that glucagon plays a key role in steroid-induced hyperglycemia, as alterations in pancreatic α-cell function led to fasting and fed hyperglucagonemia. Interestingly, the specific blockade of glucagon receptors normalized the GC-induced hyperglycemia in these rats. Additionally, we observed that hyperglucagonemia and 11βHSD-1 pre-receptor GC metabolism contribute to insulin hypersecretion.

## Methods

### Animals

The experiments were performed with 3-month-old, male Wistar rats. The rats were obtained from the animal breeding centers of Miguel Hernández University and the Federal University of Santa Catarina. They were kept at 21°C±1°C on a 12 h light/dark cycle (lights on 06:00 – lights off 18:00) and had access to commercial standard chow and water *ad libitum*. According to national regulations, the experiments were approved by the Committees for Ethics in Animal Experimentation of Miguel Hernández University (approval ID: IB-IQM-001-12) and Federal University of Santa Catarina (approval ID: PP00782).

### Dexamethasone treatment, plasma measurements and intraperitoneal glucose tolerance test [ipGTT)

Dexamethasone-treated rats (DEX) received one daily intraperitoneal (i.p.) injection (07:30–08:30 h) of dexamethasone (1 mg/kg *b.w.*) for 5 consecutive days according to previous studies [7], [13], whereas control rats (CTL) received saline (1 ml of 0.9% NaCl/kg *b.w.*). Blood was collected from the tails of fasted (12–14 h) and fed rats, and the glucose levels were measured with a glucometer (Accu-Check Performa, Roche Diagnostics GmbH, Mannhein, Germany). Euthanasia was performed by CO_2_ exposure followed by decapitation, and the trunk blood was collected in tubes containing anticoagulant and aprotinin (Sigma, Madrid, Spain) for subsequent insulin or glucagon determination. Plasma was obtained by centrifugation for 10 min at 1,000× g and 4°C, and either insulin or glucagon were measured using a Coat-a-count RIA kit (DPC, Los Angeles, CA, USA) or ELISA (Gentaur, San Jose, CA, USA), respectively, according to the manufacturer's instructions [13], [22], [23]. The homeostatic model assessment (HOMA), triacylclycerol and glucose index (TyG index) and ipGTT were performed as described previously [24]. A separate group of rats received a daily i.p. injection of des-His^1^-(Glu^9^]-glucagon (1–29) amide (1 µmol/l.kg^−1^ *b.w.*) (Tocris Bioscience, IO Centre Bristol, UK), a selective antagonist of the glucagon receptor, during the last 3 days of dexamethasone administration. This antagonist has been proven to be efficient in lowering of hyperglycemia induced by endogenous glucagon in different animal models like streptozotocin diabetic rats [25], [26].

### Islet isolation and determination of hormonal secretion

Islets were isolated by collagenase digestion as previously described [7]. The total islet insulin and glucagon contents were measured by RIA or ELISA after overnight extraction of the islets in acid-ethanol lysis buffer as previously described [23]. The protocol used to determine insulin secretion was described in detail previously [7], [13]. Briefly, after the islets were isolated, groups of five islets were incubated for 1 h at 37°C in 1 ml of a Krebs-bicarbonate buffer solution (pH 7.4) containing 5.6 mmol/l glucose supplemented with 0.05% bovine serum albumin and equilibrated with a mixture of 95% O_2_ : 5% CO_2_. The medium was then replaced with 1 ml of fresh buffer and incubated for an additional hour. Then, the supernatant was collected and stored at −80°C for the subsequent measurement of insulin. To determine glucagon secretion, groups of twelve islets were incubated for 1 h at 37°C in 0.5 ml of a Krebs-bicarbonate buffer solution containing 8.3 mmol/l glucose. The medium was then replaced with 0.4 ml of fresh buffer containing the solutions indicated in each experiment and incubated for 1 h [23]. At the end of the incubation period, the supernatants were stored at −80°C for the subsequent measurement of glucagon content by ELISA.

### Quantitative morphometric analysis of the endocrine pancreas

For the morphometric analysis, 6–7 pancreases from each group were excised and processed as previously published [12], except that 4% phosphate-buffered paraformaldehyde was used as a fixative solution. The cellular distribution of glucagon was analyzed by immunostaining with a polyclonal anti-glucagon antibody (sc7779; Santa Cruz Biotechnology, Heidelberg, Germany). The α-cell mass was determined for each immunostained pancreas section by point counting morphometry as previously described [23], with minor modifications. Briefly, each section was systematically scored with a grid of 196 points (final magnification ×200) using the image processing and analysis software ImageJ (available at http://rsbweb.nih.gov/ij/). The numbers of intercepts over α-cells, endocrine non-α-cells, exocrine pancreatic tissue, and non-exocrine pancreatic tissue were counted. The α-cell relative volume was calculated by dividing the intercepts over α-cells by the intercepts over the total pancreatic tissue; the α-cell mass was then estimated by multiplying the α-cell relative volume by the total pancreas weight. A total of 1765 and 1588 fields were counted for pancreases from control and malnourished mice, respectively.

### Transmission electron microscopy [TEM) and granule morphometry

Pools of isolated islets were processed for transmission electron microscopy as described previously [13]. Observations were made and electron micrographs were prepared using a Zeiss Leo 906 transmission electron microscope operated at 80 kV. To determine the cytoplasmic granule density, all granules were counted and divided by the cytoplasmic area. The percentage of granules docked at the plasma membrane was defined as the percentage of granules that were in contact with the plasma membrane in each median section [13]. The granules were only examined in α-cells containing a well-defined nucleus in the median section.

### Distribution of the glucagon receptor and 11βHSD-1 in the endocrine pancreas

Pancreases from each group were excised and processed as described previously [27]. Sections were cut at a thickness of 5 µm, dewaxed in xylene, and rehydrated in a graded ethanol series. The sections were incubated in PBS containing 3% BSA for 1 h at room temperature to block non-specific binding and then with the following antibodies in PBS containing 3% BSA overnight at 4°C: anti-glucagon receptor (1∶100, bs-3945R; Bioss, Woburn, MA), anti-insulin (1∶100, I2018; Sigma, Madrid, Spain), anti-glucagon (1∶100, G2654; Sigma, Madrid, Spain), and anti 11b-HSD1 (1∶50, sc-20175; Santa Cruz Biotechnology). After washing, the sections were incubated with the corresponding secondary antibodies (1∶1000, Alexa Fluor; Molecular Probes, Leiden, The Netherlands) for 1 h at room temperature. Hoechst 33342 (Life Technologies, Madrid, Spain) was used for nuclear staining. Images were captured using a Leica TCS SP2 AOBS spectral confocal microscope.

### Protein extraction and immunoblotting

Protein extraction and immunoblotting were performed as previously described [7], [8]. Pools of isolated islets were homogenized in ice-cold cell lysis buffer (Cell Signaling, Danvers, MA, USA). The protein concentration in the total cell lysate was determined by the Bradford protein assay (Bio-Rad, Hercules, CA, USA). The same amount of islet protein (50 µg) was used for each experiment. Experiments were performed at least six times using different samples (each sample consisted of islets obtained from one rat). After 2 h of blocking at RT, the membranes containing the islet protein lysates were washed with TBST and incubated overnight with the appropriate primary antibodies. The polyclonal anti-glucagon receptor (sc66912) and polyclonal anti-11bHSD-1 (sc20175) antibodies were purchased from Santa Cruz Biotechnology, and the polyclonal anti-b actin antibody (#A2066) was obtained from Sigma. After washing with TBST, the membranes were incubated with the appropriate secondary antibody. Antibody binding was detected using the Clarity Western ECL Substrate (Bio-Rad).

### Data Analysis

The results are expressed as the mean ± SEM of the indicated number (*n*) of experiments. Statistical comparisons between the data from the DEX and CTL groups were performed using the unpaired Student's t-test, with Welch correction when necessary. For unpaired groups, one-way analysis of variance (ANOVA) followed by the Tukey or Student-Newman-Keuls *post test* was used for multiple comparisons of parametric data. When necessary, the nonparametric Kruskal-Wallis test followed by the Dunn *post test* was applied. Significance was set at *p*<0.05.

## Results

### Hyperglucagonemia is involved in the increased plasma glucose levels of DEX rats

**Figure 1:**
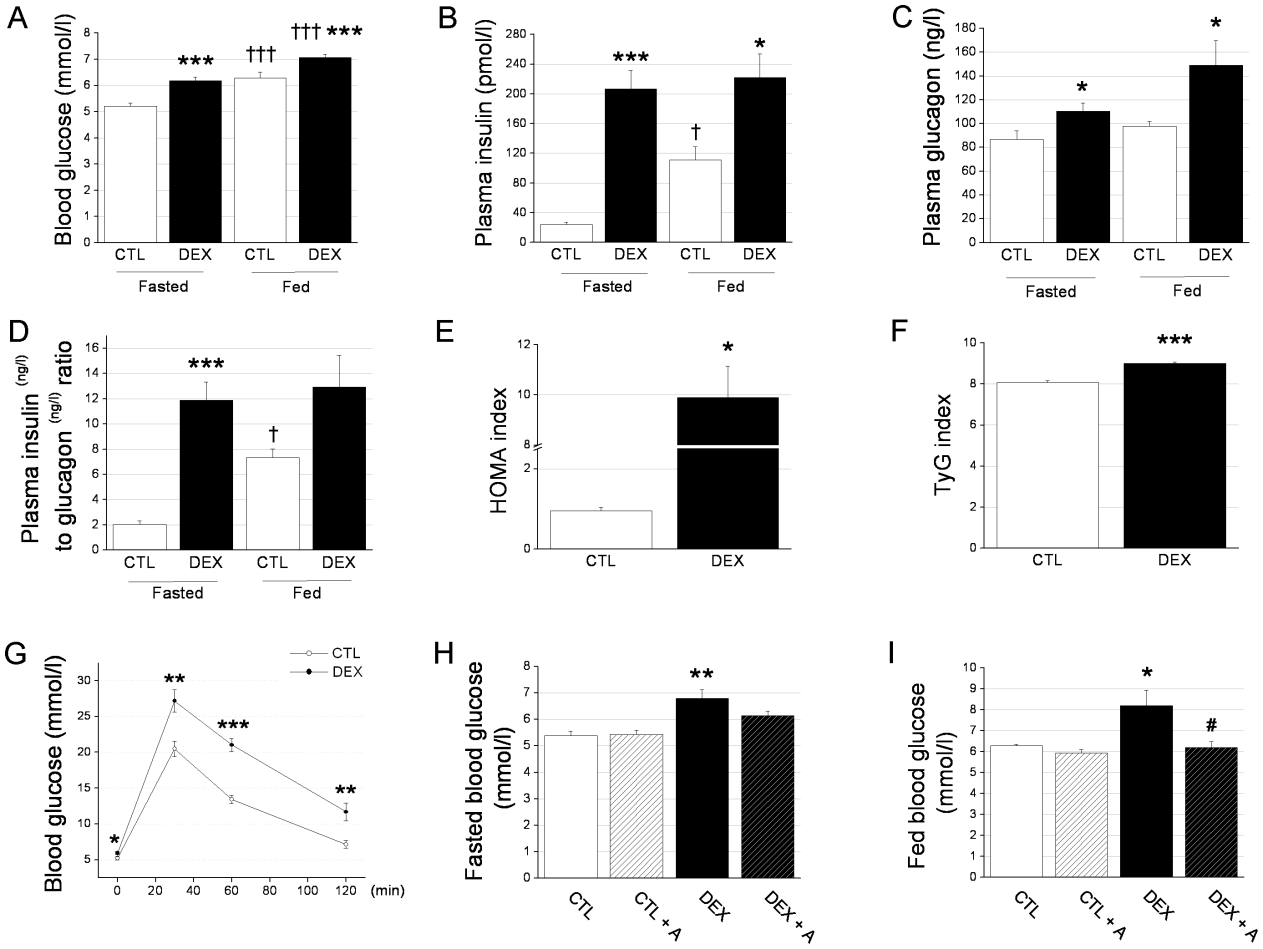
Hyperglucagonemia is involved in the hyperglycemia of DEX rats. *A*: Blood glucose, *B*: plasma insulin, *C*: plasma glucagon, and *D*: plasma insulin to glucagon ratio in fasted (12–14 h) and fed DEX and CTL rats. *E*: HOMA index, *F*: TyG index and *G*: ipGTT in fasted (12–14 h) DEX and CTL rats. *H*: Fasted and *I*: fed blood glucose in rats receiving 1 µmol/l.kg^−1^ of des-His^1^-(Glu^9^]-glucagon (1–29) amide from the third day to the last day of dexamethasone administration. Data are the mean ± SEM. *n* = 10 in *A* to *G*. *n* = 7 in *H* and *I*. * *p*<0.05, ** *p*<0.01, *** *p*<0.001 *vs.* CTL. ^†^ *p*<0.05, ^†††^ *p*<0.001 *vs.* fasted in *A*, *B*, *C* and *D*. ^#^ *p*<0.05 *vs.* DEX. HOMA, homeostatic model assessment; ipGTT, intraperitoneal glucose tolerance test.

As expected [13], GC administration caused an increase in the blood glucose concentration in both fasted (≈22%) and fed (≈21%) rats compared with the controls (Figs. 1A, G and H) as well as 8.5- and 2.0-fold increases in the plasma insulin concentration during the fasted and fed states, respectively (Fig. 1B). The plasma glucagon levels were also higher in fasted (27%) and fed (53%) rats treated with dexamethasone than rats treated with saline (Fig. 1C). The saline-treated rats had a higher plasma insulin to glucagon ratio from the fasted state to the fed state (Fig. 1D). Conversely, the plasma insulin to glucagon ratio was markedly elevated in fasted DEX rats and did not differ significantly from the fasted state to the fed state, further pointing to an impairment in the bihormonal control of glucose (Fig. 1D). In accordance with previous studies showing that GC induces insulin resistance and glucose intolerance [6], [8], [24], the HOMA and the TyG indexes and ipGTTs revealed a significant reduction in insulin sensitivity and glucose tolerance in the DEX rats compared with the control rats (Figs. 1E, F, and G, respectively). To analyze whether the elevated plasma glucagon levels were involved in the increased blood glucose concentration observed in the DEX rats, we co-treated the DEX rats with the glucagon receptor antagonist des-His^1^-(Glu^9^]-glucagon (1–29) amide [25], [26]. Under these conditions, we observed a partial attenuation of the GC-induced increase in blood glucose in the DEX rats during fasting (Fig. 1H) and a total blockage during the fed state (Fig. 1I). In summary, we found an increase in blood glucose and plasma insulin after GC treatment, which is in agreement with previous reports [7]. The hyperglucagonemia that was observed in both energetic states, the unaltered insulin/glucagon ratio from the fasted state to the fed state, and the findings from our glucagon receptor antagonist experiment suggest an important role for glucagon in the glucose homeostasis imbalance resulting from chronic GC administration.

### The inhibition of glucagon secretion by pancreatic α-cells in response to glucose is impaired in DEX rats

**Figure 2:**
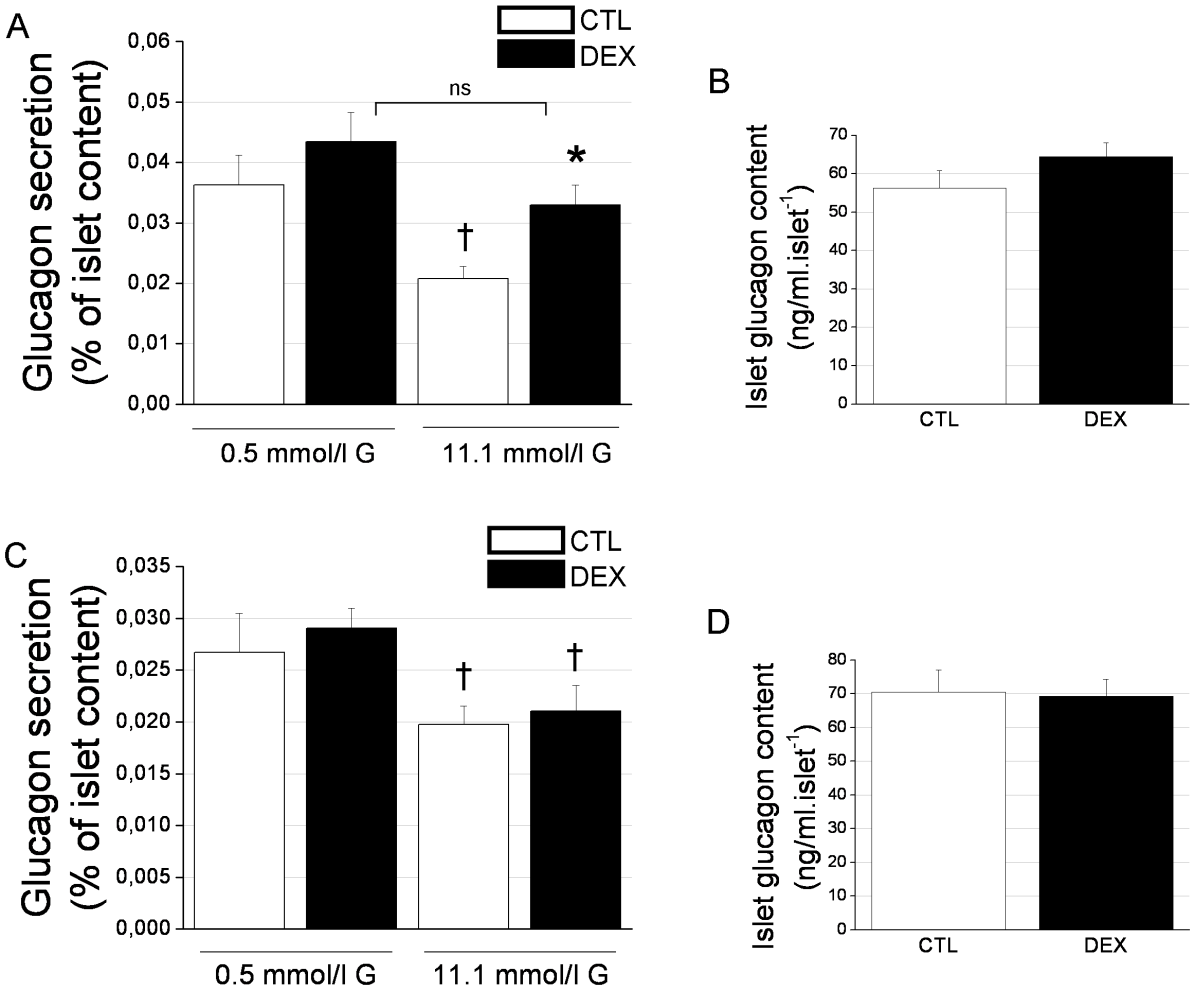
The inhibition of glucagon secretion by pancreatic α-cells in response to glucose is impaired in DEX rats. *A*: Glucagon secretion in response to 0.5 mmol/l or 11.1 mmol/l glucose in isolated islets from DEX and CTL rats (*n* = 10 wells). *B*: Total islet glucagon content (*n* = 10). *C*: *In vitro* glucagon secretion in response to 0.5 mmol/l or 11.1 mmol/l glucose in isolated islets that were pre-incubated for 3 h with 8.3 mmol glucose with or without 1 µmol/l dexamethasone (*n* = 10 wells). *D*: Total islet glucagon content (*n* = 10). Data are the mean ± SEM. * *p*<0.05 *vs.* CTL. ^†^ *p*<0.05 *vs.* 0.5 mmol/l glucose. G, glucose.

Considering the higher plasma glucagon levels found in the DEX rats, we next evaluated the pancreatic α-cell response to glucose in these animals using an *ex vivo* glucagon secretion protocol. Figure 2A shows the level of glucagon secreted after 1 h of incubation with 0.5 mmol/l or 11.1 mmol/l glucose. While 11.1 mmol/l inhibited glucagon secretion compared with 0.5 mM glucose in the control rats, as has been previously shown [17], this high level of glucose did not significantly affect the GC-treated rats (Fig. 2A). Actually, the islets of the DEX rats secreted a significantly higher amount of glucagon (57%) upon incubation with 11.1 mmol/l glucose compared with the CTL islets. The values of glucagon secretion expressed in absolute terms (ng/l) further emphasize this difference between CTL and DEX rats after incubation with 11.1 mmol/l glucose (Table 1). There was no difference in the total islet glucagon content between the DEX and CTL groups (Fig. 2B), suggesting that the differences in secretion were not related to glucagon synthesis. To assess whether dexamethasone exerted any direct effect on glucose-stimulated glucagon secretion, we performed an *in vitro* experiment with an acute 3-h preincubation of the islets with 1 µmol/l dexamethasone before the 1-h incubation period with glucose in the absence of dexamethasone. As observed in Figure 2C and Table 1, there were no differences in the glucagon response to the low or high glucose concentration between the DEX and saline-treated groups nor in the total islet glucagon content (Fig. 2D). Thus, the inhibitory effect of glucose on α-cell function is impaired in DEX rats, which may account for the augmented glucagon levels observed after GC treatment. In addition, this response does not seem to be related to increased islet glucagon content and/or direct dexamethasone effects.

**Table 1:** Glucagon secretion (ng/l) per islet in response to low or high glucose in isolated islets.

	*Ex vivo*		*In vitro*	
	0.5 mmol/l	11.1 mmol/l	0.5 mmol/l	11.1 mmol/l
**CTL**	21.0±2.7	13.0±1.7†	18.5±2.4	13.5±1.1†
**DEX**	27.8±2.9	21.4±2.2† *	20.6±1.2	14.3±1.6†

Data are expressed as mean ± SEM (*n* = 10 wells).

* *p*<0.05 *vs.* CTL and

† *p*<0.05 *vs.* 0.5 mmol/l glucose.

### The pancreatic α-cell mass in DEX-treated rats

**Figure 3:**
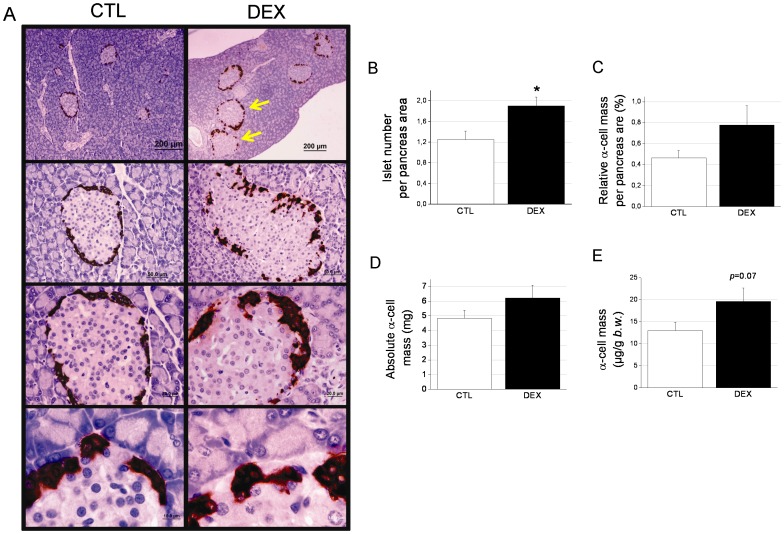
The pancreatic α-cell mass in DEX rats. *A*: Panoramic and detailed view of DEX and CTL pancreas sections that were immunostained for glucagon. *B*: Relative, *C*: absolute, and *D*: normalized α-cell mass in DEX and CTL rats. Data are the mean ± SEM (*n* = 6). Scale bars = 200, 50, 20 and 10 µm from the top to bottom images in *A*.

Changes in the pancreatic α-cell mass may contribute to alterations in the plasma glucagon level. In Figure 3A, we show a panoramic view of several pancreas sections. Note the typical hypertrophied islets in the DEX pancreas sections (arrows in Fig. 3A) as a result of β-cell hyperplasia and hypertrophy [12]. There was no apparent change in the proportion of α-cells per islet between the treatment groups (22.3±1.7% and 21.4±2.7% for the CTL and DEX rats, respectively). The islet density (number per pancreas area) was significantly higher in pancreas from DEX rats (*p*<0.05) (Fig. 3B). The relative and absolute α-cell mass was larger in the DEX pancreases compared with the CTL pancreases (69% and 28%, respectively), although these differences were not statistically significant (Figs. 3C and D). When the absolute α-cell mass was normalized to the body weight, we found a 51% increase in the DEX group compared with the control group (*p* = 0.07) (Fig. 3E). The pancreas masses values were 1.1±0.05 g for CTL and 0.9±0.04 g for DEX rats, whereas the average body weights were 328±5 and 287±4 g for CTL and DEX, respectively. As expected [7], [12], the non-α-cell mass, which is predominantly comprised of β-cells, was higher in the DEX rats compared with the controls (Figure S1). Thus, in addition to the alterations in α-cell function, an increased α-cell mass, although not statistically significant, might play a role in the excessive glucagon levels found in the GC-treated rats.

### The α-cell ultrastructure indicates an increase in cell function and docked glucagon granules at the cell membrane in DEX rats

**Figure 4:**
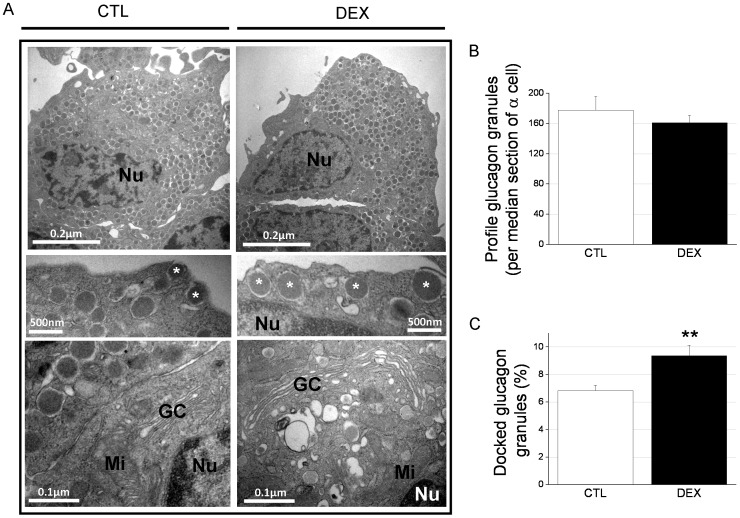
The number of docked glucagon granules is increased in the α-cells of DEX rats. *A*: Electron micrographs of α-cells from CTL and DEX rats. The images demonstrate an enlargement of the Golgi complex (GC) and the increase in glucagon granules docked to the plasma membrane in the DEX α-cells (*). N, Nucleus; Mi, mitochondria. *B*: Profile of glucagon granules per median section of α-cells. *C*: Proportion of granules docked to the plasma membrane of α-cells. Data are the mean ± SEM (*n* = 16 cells in each condition). ** *p*<0.01 *vs.* CTL.

Considering our evidence of increased α-cell function in the DEX rats, we next investigated the ultrastructural characteristics and organelle distribution of the α-cells. The main ultrastructural change observed in the DEX rat α-cells was an enlargement of the cisternal space in the Golgi complex (Fig. 4A). Quantification of the total granule number in the α-cells per median section revealed no change between the treatment groups (Fig. 4B). The glucagon granule density in the cytosol was not altered between the DEX and CTL rats (data not shown). However, the proportion of glucagon granules docked to the cell membrane was significantly increased in the α-cells in the DEX rats compared with controls (Fig. 4C). These data support the enhancement of α-cell secretory function in DEX rats.

### Glucagon is involved in the hypersecretion of insulin by the islets of DEX rats

**Figure 5:**
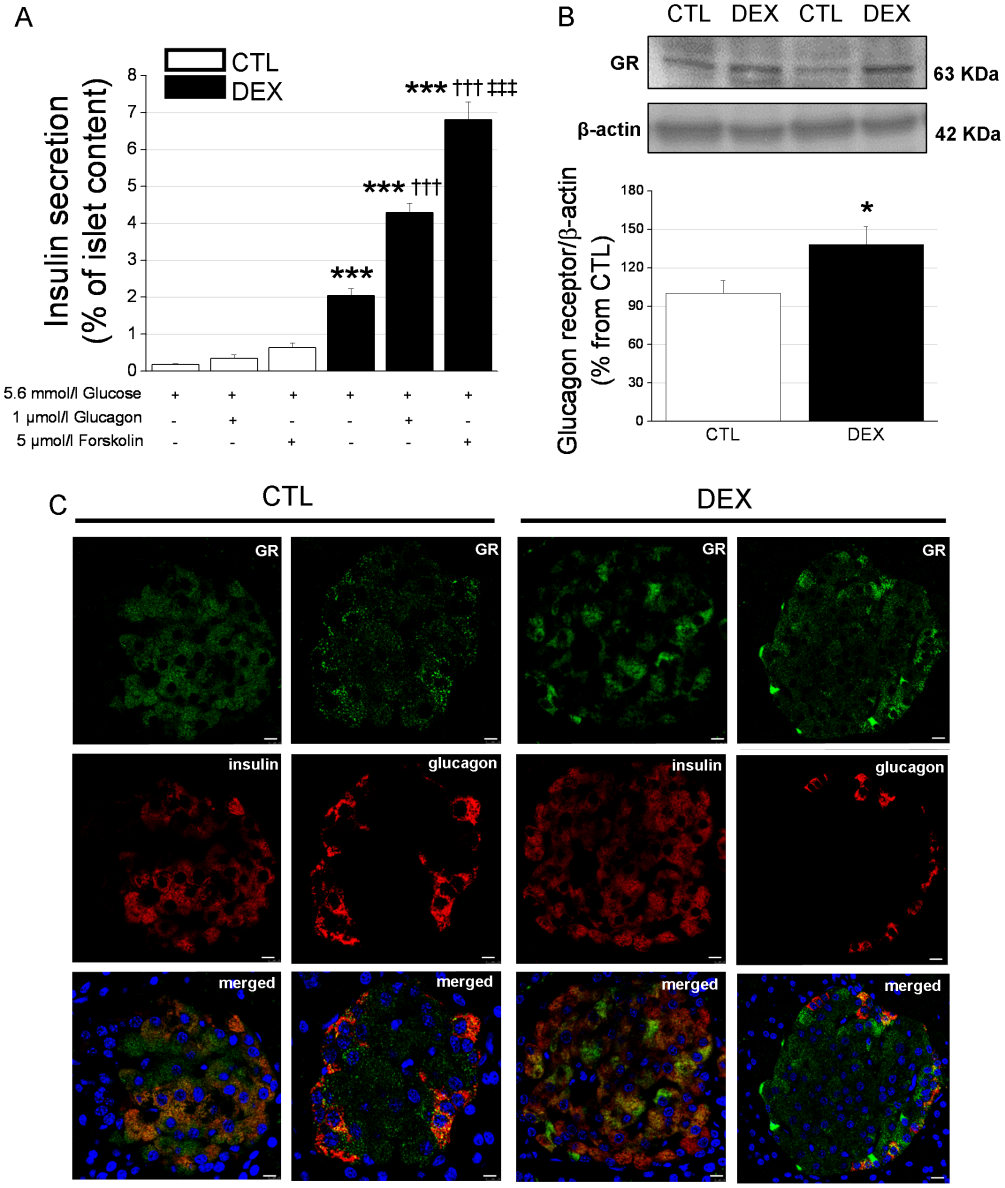
Glucagon stimulates insulin secretion in islets isolated from DEX rats. *A*: Insulin secretion in response to incubation with 5.6 mmol/l glucose with or without glucagon or forskolin in islets isolated from DEX and CTL rats (*n* = 10 wells). *B*: Representative immunoblots for the glucagon receptor (GR) and β-actin and their quantification (bar graphs) for DEX and CTL islet lysates (*n* = 6 independent samples). Data are the mean ± SEM. * *p*<0.05, *** *p*<0.001 *vs.* CTL. ^†††^ *p*<0.001 *vs.* 5.6 m glucose. ^‡‡‡^ *p*<0.001 *vs.* 5.6 mmol/l glucose plus 1 µmol/l glucagon in *A. C*: Immunostaining of pancreas sections from DEX and CTL rats for the GR (green) and insulin or glucagon (red). Merged in orange. DAPI was used for nuclei staining (*n* = 3 pancreases). Scale bars = 7.5 µm.

Considering the marked increase in the plasma insulin concentration under fasting conditions in DEX rats and that insulin secretion may be positively modulated by glucagon action [20], [21], we next investigated whether glucagon exerts a higher impact on the β-cell response to basal glucose concentrations. In Figure 5A, we show the insulin response to 5.6 mmol/l glucose alone or in combination with 1 µmol/l glucagon or 5 µmol/l forskolin. Incubation of islets from CTL rats in the presence of glucagon or forskolin did not result in a significant increase in insulin secretion (Figure 5A and Table 1). In contrast, for the islets of DEX rats, the insulin response in the presence of glucagon or forskolin was significantly higher compared to incubation with 5.6 mmol/l glucose alone (Figure 5A and Table 2). This insulin hypersecretion in DEX animals seems not to be related with increased insulin biosynthesis since the total islet insulin content was slightly decreased in DEX rats (2.48±0.05 *vs.* 2.8±0.06 nmol/l.islet^−1^ for DEX and CTL groups, respectively). Thus, glucagon seems to exert a positive modulatory effect on insulin secretion under basal glucose concentrations in DEX rats, and this action may be involved in the fasting hyperinsulinemia observed after GC treatment.

**Table 2:** Insulin secretion ratio in isolated islets from CTL and DEX rats.

	CTL	DEX
**Glucagon/Glucose**	1.6±0.2	2.3±0.2*
**Forskolin/Glucose**	3.2±0.7	3.5±0.5

The medium containing 1 µmol/l glucagon or 5 µmol/l forskolin also contained 5.6 mmol/l glucose. Data are expressed as mean ± SEM (*n* = 10 wells).

* *p*<0.05 *vs.* CTL.

Western blot analysis revealed that the DEX rats had a higher (38%) level of glucagon receptor in their islets (Fig. 5B), further supporting the stimulatory effect of glucagon on insulin secretion upon GC treatment. Immunocytochemistry of pancreas sections showed the localization of the glucagon receptor in the pancreatic β-cells (as well as in α-cells) in both groups (Fig. 5C).

### Involvement of 11βHSD-1 in insulin secretion

**Figure 6:**
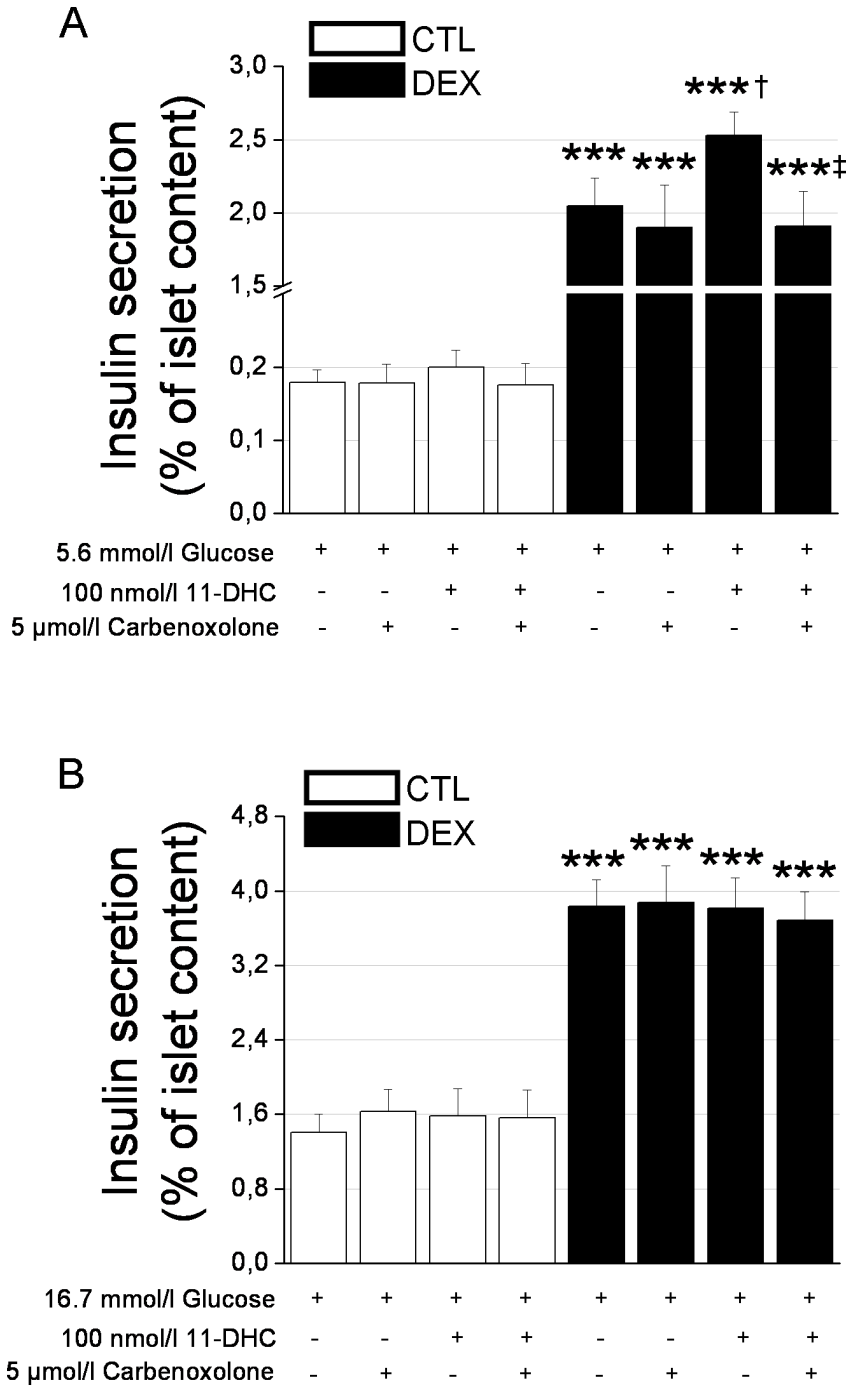
11-Dehydrocorticosterone [11-DHC) stimulates insulin secretion under basal glucose conditions in islets isolated from DEX rats. *A*: Insulin secretion in response to incubation with 5.6 mmol/l glucose with or without 11-DHC or carbenoxolone in islets isolated from DEX and CTL rats (*n* = 10 wells). *B*: Insulin secretion in response to incubation with 16.7 mmol/l glucose with or without 11-DHC or carbenoxolone in islets isolated from DEX and CTL rats (*n* = 10 wells). Data are the mean ± SEM. *** *p*<0.001 *vs.* CTL. ^†^ *p*<0.05 *vs.* 5.6 mmol/l glucose. ^‡^ *p*<0.05 *vs.* 5.6 mmol/l glucose plus 100 nmol/l 11-DHC.

Because inactive endogenous 11-DHC is locally converted to active corticosterone (CORT) inside islets by 11βHSD-1 and because CORT modulates insulin secretion, we next evaluated the effect of 11-DHC on β-cell function. As expected [7], [11], insulin secretion in response to 5.6 mmol/l or 16.7 mmol/l glucose was significantly higher in the islets of the DEX rats compared to those of controls (Figs. 6A and B). Incubation with 11-DHC and 5.6 mmol/l glucose showed no impact on insulin secretion in the CTL rats but resulted in higher insulin release in the DEX rats. The insulin increase observed in the presence of 11-DHC was totally abrogated by co-incubation with the 11βHSD-1 antagonist carbenoxolone (5 µmol/l). The effect of 11-DHC in the presence of 5.6 mmol/l glucose was not observed with islets challenged with 11-DHC and 16.7 mmol/l glucose for both treatment groups (Fig. 6B), most likely because this glucose concentration is close to the maximal stimulation of insulin release. Altogether, these data provide evidence for a role of pre-receptor glucocorticoid metabolism in insulin secretion under basal physiological conditions that supports insulin hypersecretion and fasting hyperinsulinemia.

**Figure 7:**
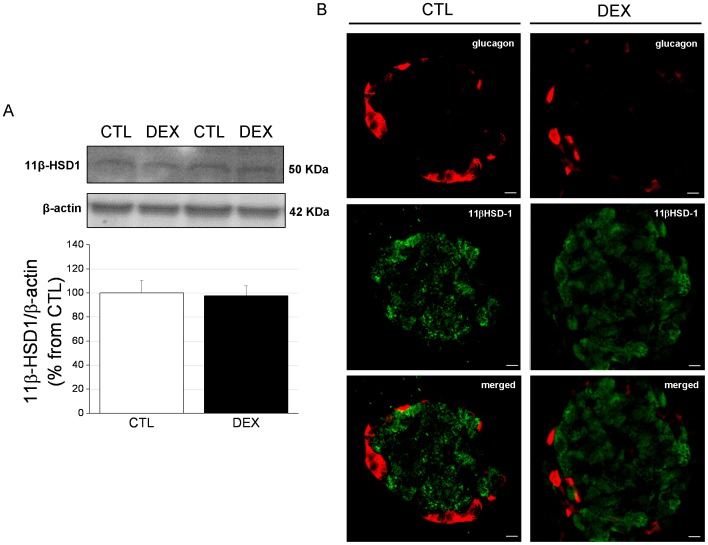
11β Hydroxysteroid dehydrogenase type 1 [11βHSD-1) content and cellular distribution in the pancreas in DEX rats. *A*: Representative immunoblots for 11βHSD-1 and β-actin and their quantification (bar graphs) in DEX and CTL islet lysates (*n* = 6 independent samples). Data are the mean ± SEM. *C*: Immunostaining of pancreas sections from DEX and CTL rats for glucagon (red) and 11βHSD-1 (green). Merged in orange. *n* = 3 pancreases. Scale bars = 7.5 µm.

No differences were observed in the 11βHSD-1 protein content between the DEX and CTL groups (Fig. 7A). In contrast with a previous report showing that 11βHSD-1 is predominantly located in glucagon-containing cells [28], we did not observe co-localization of this enzyme with the peripheral pancreatic α-cells (Fig. 7B). Instead, 11βHSD-1 was detected in the cells located in the islet core, which were most likely β-cells.

## Discussion

The classical adverse effects of GC therapy on glucose and nutrient metabolism have been well known for decades. They include glucose intolerance, peripheral insulin resistance, elevation of the blood glucose levels, hyperinsulinemia, and alterations in pancreatic β-cell function as our group and others have previously reported [2], [3], [8], [12], [13]. The involvement of pancreatic α-cells in this phenomenon has now emerged and raises new questions related to the mechanisms underlying the imbalance in glucose homeostasis caused by GC treatment. Here, we show for the first time that glucose-intolerant and insulin-resistant rats created by dexamethasone treatment exhibit 1) fasting and fed hyperglucagonemia, 2) an unaltered insulin/glucagon ratio from the fasted state to the fed state, 3) impaired glucose-induced suppression of glucagon secretion, 4) a trend to increase in α-cell mass, 5) an increase in the number of docked glucagon granules 6) augmented islet glucagon receptor content as well as insulin secretion in response to glucagon, and 7) enhanced insulin secretion in response to 11-DHC under basal glucose conditions. The significant reduction in the blood glucose levels in DEX rats by treatment with a glucagon receptor antagonist points to the crucial involvement of glucagon in the diabetogenic effects of GCs and also indicates the antagonism of the glucagon receptor as a potential therapeutic target to ameliorate the hyperglycemia resulting from GC therapy.

In the 1970s, it was shown that non-obese human subjects treated with dexamethasone developed high plasma glucose and insulin levels along with increased fasting plasma glucagon levels [14]. These individuals also showed a higher glucagon response to alanine infusion compared to non-obese individuals. More recently, altered fasting glucagon levels were observed in rhesus macaques after dexamethasone treatment [18] and in rats by the combination of exogenous corticosterone and high-fat diet [29]. Hyperglucagonemia and impaired α-cell function have been linked to the etiology of both T1DM and T2DM [17]. Under these pathological conditions, high glucose levels fail to suppress glucagon release, favoring higher plasma glucagon levels, which promote hepatic glucose output and contribute to hyperglycemia [17]. In the present study, DEX rats exhibited high plasma glucagon concentrations under both fasted and fed conditions. Interestingly, their insulin/glucagon ratio did not increase from the fasted state to the fed state, which may result in impaired insulin suppressive effects and/or augmented hyperglycemic actions of glucagon on the liver, as was previously suggested for GC-treated humans [30]. In fact, the DEX rats are less insulin sensitive as observed by the insulin tolerance test and basal adipose tissue glycerol release as previously reported [7], [8], [24]. The absence of increment in the insulin/glucagon ratio may also indicate a relatively poor insulin secretion under feeding as confirmed by the GTT experiments (Fig. 1G) in the DEX rats. The involvement of glucagon in the high blood glucose levels of DEX rats was supported by experiments with a glucagon receptor antagonist, which fully prevented the elevation in the glycemic values of fed DEX rats. Previous data with the same antagonist have showed its inability to activate glycogenolysis as well as its ability to lowers the hyperglycemia produced by endogenous glucagon in streptozotocin diabetic rats [30]. Herein, the partial effects of the antagonist in attenuating the blood glucose level in the fasted state point to the presence of other altered circulating factors that may act in synergy with glucagon. Thus, hyperglucagonemia contributes to the impaired glucose homeostasis of GC-treated rats despite their markedly increased plasma insulin concentration.

Glucagon secretion in response to high glucose concentrations was not properly suppressed in the islets of DEX rats compared with the controls. This phenomenon was not associated with altered glucagon synthesis or direct effects of dexamethasone on α-cells. In agreement with our findings, mice pretreated with prednisolone show enhanced α-cell secretory activity in response to arginine, which is not associated with changes in the islet glucagon content or a direct effect of prednisolone [31]. It has been proposed that glucagon hypersecretion in the presence of high glucose concentrations, as in T2DM, may be associated with a lower inhibitory effect of insulin on α-cells [32], impaired glucose sensing by α-cells or malfunction of neural regulation [17] rather than augmented α-cell expansion [16]. The fact that glucagon release did not change in response to low, but did in response to high glucose indicates that these values are primarily consequence of impaired function. Interestingly, we found that in DEX rats, the glucagon levels were higher under conditions of augmented blood glucose levels and hyperinsulinemia. Notwithstanding, the amount of α-cell mass may not be neglected in DEX rats, since the non-significant increase (∼51%) of the normalized α-cell mass may contribute for the increased circulating glucagon levels. Mice lacking microRNA-375 (375KO) share some characteristics with our DEX rats (*e.g.*, hyperglycemia, fast and fed hyperglucagonemia, glucose intolerance and reduced effect of glucose to inhibit glucagon secretion) [33]. The authors associated the involvement of α-cells with the disrupted glucose homeostasis based on the 29% increase of the relative α-cell (no data regarding the absolute α-cell mass was shown). Thus, several processes, whose molecular basis merits further investigation, may contribute to excessive glucagon release.

Numerous studies with mice [20], rats [21], [34] and humans [26] have reported the presence of functional glucagon receptors in β-cells. Glucagon generates synergic signals in glucose-stimulated insulin secretion (GSIS) [20], [21] that involve the activation of adenylyl cyclase and protein kinase A (PKA). Here, we observed that islets from DEX rats secreted more insulin in response to glucagon at basal glucose conditions compared with islets from control rats. This insulin hypersecretion might be involved in the marked 8.5-fold increase in the fasted plasma insulin values, among other factors (*e.g.*, highly islet insulin response to glucose) [7]. This effect was only significant in the islets from the DEX rats but not in the islets from the controls, which reinforces the role for glucagon in β-cells in DEX rats. These findings point to an apparent increase in glucagon sensitivity in the islets of DEX rats. Consistent with this idea, the islets from DEX rats contained higher levels of glucagon receptor. Additionally, forskolin, an activator of adenylyl cyclase, was more potent in the islets from DEX rats at 5.6 mmol/l glucose. Insulin secretion may also be modulated by intra-islet GC metabolism. It was previously demonstrated that the enzymatic activity of 11βHSD-1, which generates active corticosterone from inactive 11-DHC, results in a reduction in insulin secretion *in vitro* [28]. In *in vivo* conditions, however, the adequate elevation of β-cell 11βHSD-1 activity is a compensatory mechanism that prevents high-fat diet-induced β-cell failure [35]. In addition, there is evidence that volunteers treated with prednisolone for 6 consecutive days have increased 11βHSD-1 activity, as the administration of cortisone in these individuals results in a marked rise in the serum cortisol level and the cortisol/cortisone ratio [36]. Islets from DEX rats, but not from control rats, secreted more insulin in response to 5.6 mmol/l glucose in the presence of 11-DHC. While the elevation of 11βHSD-1 activity in the hepatic and adipose tissue leads to disturbances in glucose metabolism, mimicking metabolic syndrome [37], our data indicate that, at least for islet function, intra-islet GC metabolism may be involved in the compensatory insulin hypersecretion that is required to face the GC-imposed IR in DEX rats. These findings are consistent with previous observations [35].

In conclusion, treatment with high doses of GC induces hyperglucagonemia, which disrupts glucose homeostasis. The impairment of the α-cell response to inhibitory glucose signals agrees with the increase in plasma glucagon, which, in parallel, may contribute to the compensatory β-cell hypersecretion resulting from GC therapy. Blockage of the glucagon receptor seems to be effective in preventing GC-induced hyperglycemia and represents a potential mechanism for the treatment of hyperglycemia induced by GC treatment.

## Supporting Information

Figure S1 **Morphometric analysis of non-α cells.** *A*: Relative, *B*: absolute, and *C*: normalized non-α-cell mass in DEX and CTL rats. Data are the mean ± SEM (*n* = 6). * *p*<0.05 *vs.* CTL.
(TIF)
