# Supplementary figures and images for: Pancreatic Alpha-Cell Dysfunction Contributes to the Disruption of Glucose Homeostasis and Compensatory Insulin Hypersecretion in Glucocorticoid-Treated Rats
Source: PLoS One. 2014 Apr 4;9(4):e93531. doi: 10.1371/journal.pone.0093531 (PMC3976288; doi:10.1371/journal.pone.0093531)

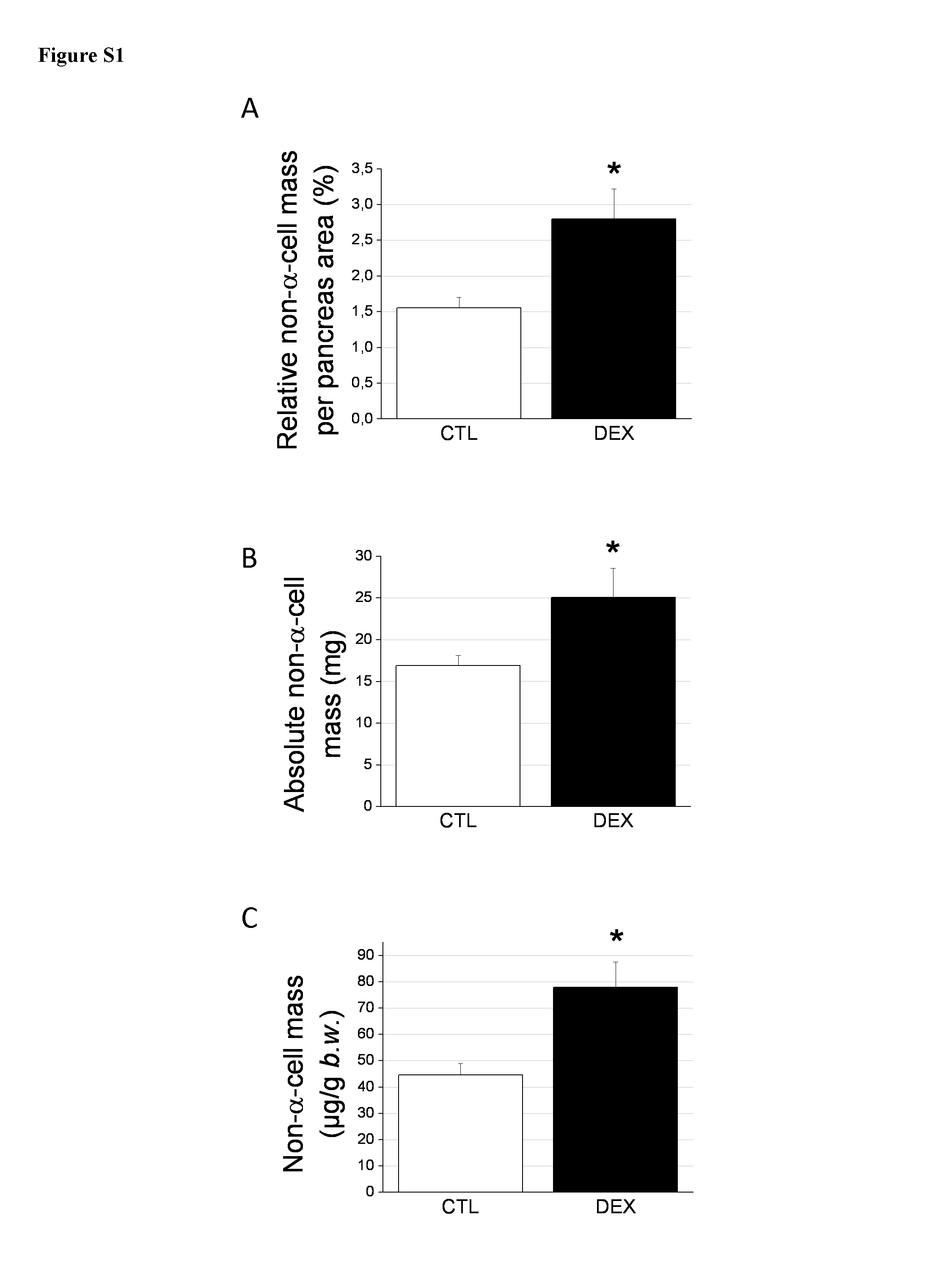

Supplement: Figure S1 — Morphometric analysis of non-α cells. A: Relative, B: absolute, and C: normalized non-α-cell mass in DEX and CTL rats. Data are the mean ± SEM (n = 6). * p<0.05 vs. CTL. (TIF) [file pone.0093531.s001.tif]
